# The Frontal Aslant Tract: A Systematic Review for Neurosurgical Applications

**DOI:** 10.3389/fneur.2021.641586

**Published:** 2021-02-24

**Authors:** Emanuele La Corte, Daniela Eldahaby, Elena Greco, Domenico Aquino, Giacomo Bertolini, Vincenzo Levi, Malte Ottenhausen, Greta Demichelis, Luigi Michele Romito, Francesco Acerbi, Morgan Broggi, Marco Paolo Schiariti, Paolo Ferroli, Maria Grazia Bruzzone, Graziano Serrao

**Affiliations:** ^1^Department of Neurosurgery, Fondazione IRCCS (Istituto di Ricovero e Cura a Carattere Scientifico) Istituto Neurologico Carlo Besta, Milan, Italy; ^2^San Paolo Medical School, Department of Health Sciences, Università degli Studi di Milano, Milan, Italy; ^3^Neuroradiology Department, Fondazione IRCCS (Istituto di Ricovero e Cura a Carattere Scientifico) Istituto Neurologico Carlo Besta, Milan, Italy; ^4^Department of Neurological Surgery, University Medical Center Mainz, Mainz, Germany; ^5^Parkinson's Disease and Movement Disorders Unit, Department of Clinical Neurosciences, Fondazione IRCCS (Istituto di Ricovero e Cura a Carattere Scientifico) Istituto Neurologico Carlo Besta, Milan, Italy; ^6^Department of Health Sciences, Università degli Studi di Milano, Milan, Italy

**Keywords:** diffusion-weighted imaging, executive function skills, frontal aslant tract, language, working memory, motor coordination, neurosurgery, tractography

## Abstract

The frontal aslant tract (FAT) is a recently identified white matter tract connecting the supplementary motor complex and lateral superior frontal gyrus to the inferior frontal gyrus. Advancements in neuroimaging and refinements to anatomical dissection techniques of the human brain white matter contributed to the recent description of the FAT anatomical and functional connectivity and its role in the pathogenesis of several neurological, psychiatric, and neurosurgical disorders. Through the application of diffusion tractography and intraoperative electrical brain stimulation, the FAT was shown to have a role in speech and language functions (verbal fluency, initiation and inhibition of speech, sentence production, and lexical decision), working memory, visual–motor activities, orofacial movements, social community tasks, attention, and music processing. Microstructural alterations of the FAT have also been associated with neurological disorders, such as primary progressive aphasia, post-stroke aphasia, stuttering, Foix–Chavany–Marie syndrome, social communication deficit in autism spectrum disorders, and attention–deficit hyperactivity disorder. We provide a systematic review of the current literature about the FAT anatomical connectivity and functional roles. Specifically, the aim of the present study relies on providing an overview for practical neurosurgical applications for the pre-operative, intra-operative, and post-operative assessment of patients with brain tumors located around and within the FAT. Moreover, some useful tests are suggested for the neurosurgical evaluation of FAT integrity to plan a safer surgery and to reduce post-operative deficits.

## Introduction

Refinements in the study of the human brain white matter by different means, such as dissection and advanced MR imaging techniques are leading to the discovery of new brain pathways. The frontal aslant tract (FAT) is a brain white matter tract connecting the superior frontal gyrus (SFG), specifically the pre-supplementary motor area (pre-SMA), supplementary motor area (SMA), and lateral SFG to the pars opercularis and pars triangularis of the inferior frontal gyrus (IFG) and the anterior insula. The first time that connectivity between the pre-SMA and the IFG was established was in 2007 (1). Catani et al. (2) and Thiebaut de Schotten et al. (3) were the first to explicitly name the FAT because of its oblique direction within the frontal lobe. Since then, the FAT has been described using *ex vivo* fiber dissections (4–14). Although from the discovery of such white matter tract many papers described its role in different functions, such as speech and language functions (15–18), working memory (19–21), and visual–motor activities (22–25), and its possible involvement in the pathogenesis of several neurological, psychiatric, and neurosurgical disorders, the awareness of such fascicle is still not well-popularized in the neurosurgical community. For this reason, we decided to perform a systematic literature review and to focus on the neurosurgical applications of the current knowledge on the FAT. Our objective is to suggest practical indications and useful tests for the pre-operative, intra-operative, and post-operative evaluation of patients with brain tumors located around and within this tract or patients undergoing frontal lobe epilepsy surgery, providing to the neurosurgeon useful information to plan a safer surgery and to reduce post-operative deficits.

## Methods

### Search Strategy

We performed a systematic review according to the Preferred Reporting Items for Systematic Reviews and Meta-analyses (PRISMA) statement guidelines (26). We used the following databases for the search: PubMed, Ovid MEDLINE, and Ovid EMBASE. We used the terms “FRONTAL,” “ASLANT,” and “TRACT” as individual keywords or MeSH terms in combination with the Boolean operator “AND” to maximize the identification of articles describing the FAT. Full search strategies are detailed for each database as follows: the PUBMED query was (“frontal”[All Fields] OR “frontalis”[All Fields] OR “frontalization”[All Fields] OR “frontally”[All Fields] OR “frontals”[All Fields]) AND “aslant”[All Fields] AND (“tract”[All Fields] OR “tracts”[All Fields]), and the Ovid MEDLINE and EMBASE queries were “frontal” AND “aslant” AND “tract.” The search was conducted including all the articles published until 31 July 2020, and no restrictions were applied for the study design.

Data were extracted by two independent authors (DE and EG) and reviewed by a third author (ELC). The results were exported to the Mendeley citation manager, and after duplicate removal, title and abstracts were firstly screened and full text were obtained. The reference lists of the full-text papers were examined to identify additional relevant studies. Any dissension was resolved through discussion between the three independent reviewers, and an agreement was reached on all the articles included in the review.

### Selection Criteria

The selection criteria applied to the systematic review were the following: studies written in English language involving human participants (only animal studies were excluded) and investigating brain white matter through post-mortem dissection or *in vivo* brain imaging techniques. Studies were excluded if they were not published as a full text in English because of insufficient data. During full-text screening, 19 articles were further excluded, including five reviews not introducing new concepts.

### Data Collection

Data from the included articles were extracted, assembled, and analyzed using Microsoft Excel 2019 (Microsoft Corp, Redmond, WA). The details collected consisted of the study title, authors, first author's country, publication year, publication journal, type of research (anatomical, clinical, or surgical), subjects (patient or human cadaver), total population sample size, pathology investigated, and the main result of the study.

## Results

**Figure 1:**
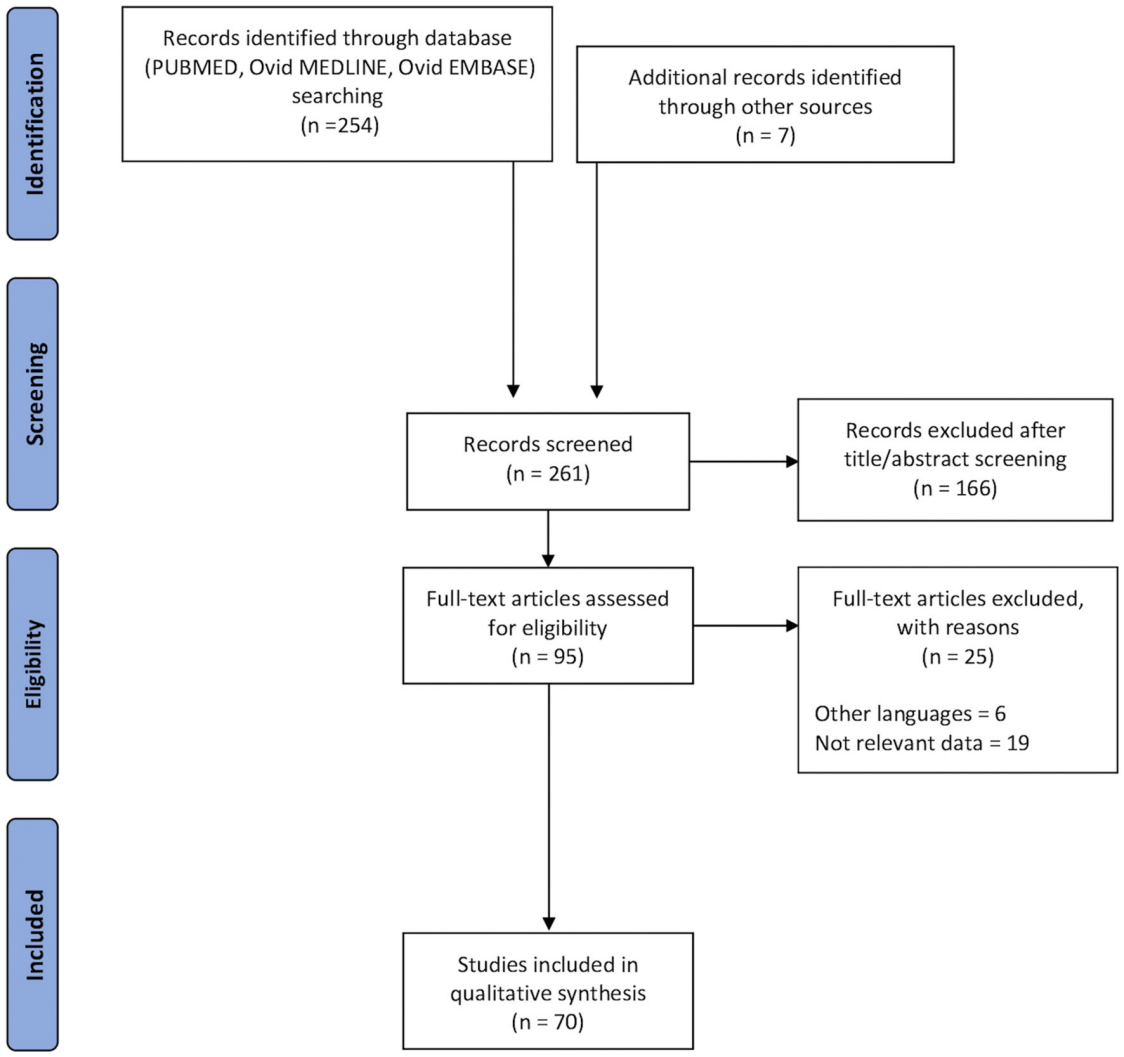
Flow chart applied to the retrieval and selection of studies included in the systematic literature review according to PRISMA guidelines.

A total of 261 records were retrieved (Figure 1). After 166 duplicate records have been removed, the titles and abstracts of 95 records were screened. During exclusion criteria application and full-text screening, 25 records were excluded, with 70 remaining articles from 2012 to July 2020, including anatomical, clinical, and neurosurgical studies. To review the available data about the FAT, we started describing the anatomy and then we highlight its role in different brain function fields, such as language, executive functions, lexical decisions, stuttering, oro-facial movements, working memory, social community tasks, attention, and music processing.

## Discussion

### Anatomy

#### Cortical Connections

**Figure 2:**
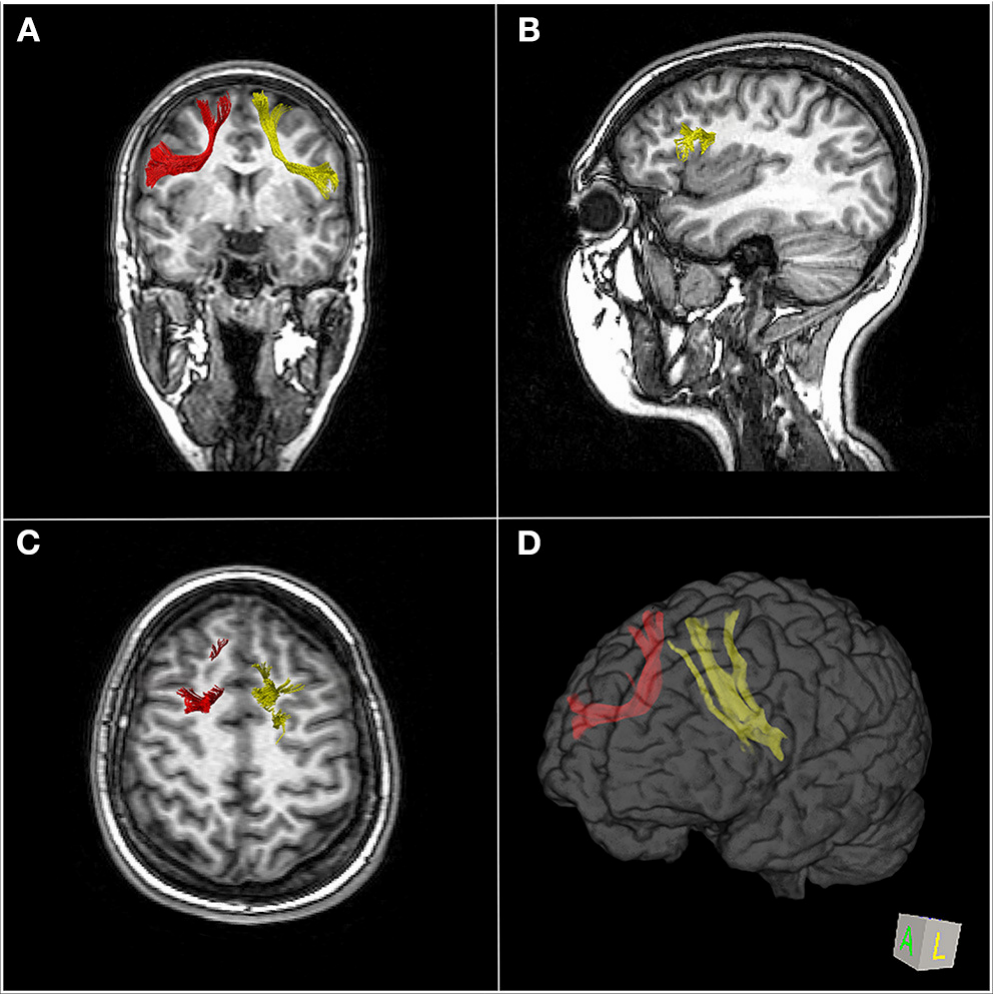
MR-diffusion tensor imaging of the frontal aslant tract (FAT) in a 23-year-old healthy female. Right (red) and left (yellow) FAT overlaid over coronal images **(A)**, left FAT terminations in the left posterior inferior frontal gyrus overlaid over sagittal images **(B)**, superior right and left FAT terminations, respectively, in the right and left superior frontal gyrus overlaid over axial images **(C)**. 3D brain reconstruction of right and left FAT **(D)**.

The FAT is a white matter fiber tract traveling in the coronal plane connecting the SFG to the ipsilateral IFG (27) (Figure 2). According to the parcellation scheme developed by the Human Connectome Project (HCP), the FAT connects the SFG, in particular, two parcellations of the SMA complex (6ma and SFL) and two of the dorsolateral prefrontal cortex (8BL and S6-8) to the IFG (parcellations 44, 6r) and the frontal operculum (parcellations FOP1, FOP3, and FOP4) as well as the middle insula (MI) parcellation in the anterior insula (28, 29). In line with the parcellation scheme, the tractography of the FAT shows terminations into the SFG, including not only the pre-SMA and SMA but also the lateral SFG (30). Varriano et al. (21) defined the extended FAT, “exFAT,” as the FAT projecting more anteriorly into the SFG. Catani et al. (15) reported the termination of the FAT into the anterior cingulate cortex. The major projection of the FAT in the IFG is the pars opercularis, but some fibers may also reach the pars triangularis (2, 27) and the inferior region of the pre-central gyrus (PrCG) (2). Non-homologous callosal connections have been described between the premotor areas, and some authors introduced the concept of “crossed FAT” that may have a role in the recovery from the SMA syndrome (10).

In children, the predominance of fibers that travel from the IFG-pars opercularis (IFG-Op) projects to the pre-SMA, but projections to the SMA and to the anterior cingulate are also found (31).

An anterior and posterior component of the FAT have been described, the first connecting Brodmann area (BA) 44 with the pre-SMA, and the latter connecting BA 6 with the SMA (17). Conflicting evidence about volumetric lateralization could be found in the current literature. While some papers suggested a left lateralization of the FAT in right-handed individuals (3), other studies found no trend of lateralization across 29 (32) and 10 healthy subjects (4, 33). In 19 typical 5- to 8-year-olds children, the FAT showed right laterality and a trend toward increasing left laterality with age (31). Variable age-related changes in the microstructure were noticed until early adulthood (31, 33, 34).

The presence of a bidirectional connection between the SFG to the Broca area has also been demonstrated through corticocortical evoked potentials (CCEPs). The latencies of CCEP responses were significantly shorter in the SFG from the Broca area stimulation than in the Broca area from the SFG stimulation (35). This could be explained by the presence of a direct corticocortical pathway from the Broca area to the SFG and an indirect cortico-subcortical pathway connecting the SFG to the Broca area. Another explanation is that different latencies reflect antidromic or orthodromic projection (35).

#### Superior Frontal Gyrus

**Figure 3:**
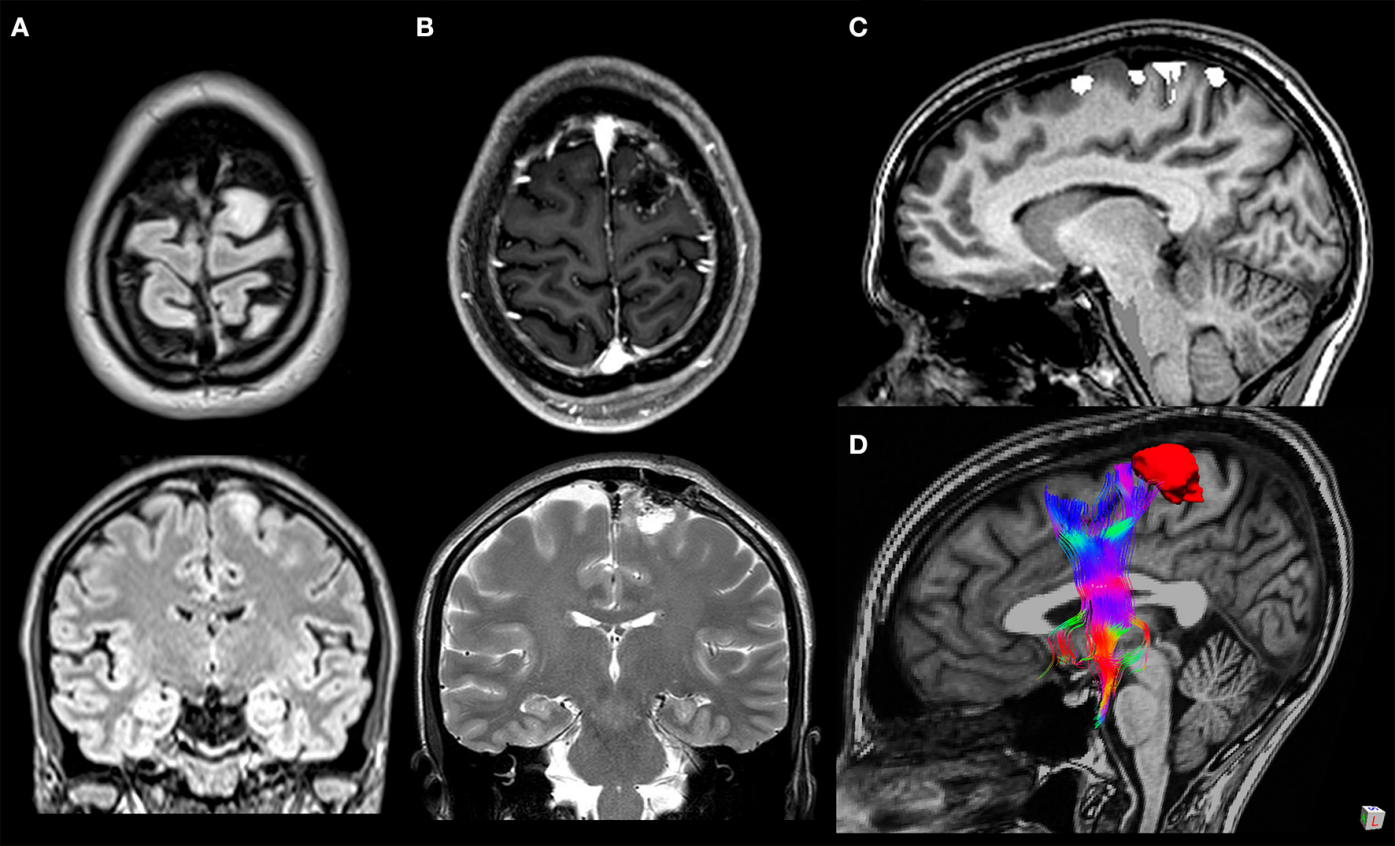
A 33-year-old woman with a WHO grade II astrocytoma located in the left cortico-subcortical region of the superior frontal gyrus. Surgical resection was performed through fluorescein-guided microsurgical technique guided by intraoperative neurophysiological monitoring and by functional MRI (fMRI)/tractography-integrated neuro-navigation system. Pre-operative symptoms included motor partial seizure affecting the right leg, followed by a generalized seizure. The patient post-operatively developed a transient mild weakness in the right leg. Preoperative axial and coronal T2-weighted MR images **(A)**, post-operative axial T1-weighted post-gadolinium and coronal T2-weighted MR images **(B)**, fMRI with blood oxygen level-dependent response in the left paracentral lobule evoked during voluntary movement of the right foot overlaid on sagittal T1-weighted MR images **(C)**, and 3D relationship between the tumor and frontal aslant tract tractography reconstruction **(D)**.

The terminations of the FAT are still objects of study. The upper terminations are commonly identified in the SMA complex in the medial SFG, but also in the dorsolateral prefrontal cortex of the SFG (28–30). The SMA complex is subdivided into the SMA proper, the pre-SMA anteriorly and the supplementary eye field (9, 30) both in the medial surface of the SFG (30), delimitated superiorly by the superior hemispheric border, the cingulate sulcus inferomedially, and the precentral sulcus posteriorly (36) (Figure 3). The anterior border of the pre-SMA is an imaginary line tangential to the rostral portion of the corpus callosum genu and perpendicular to the line connecting the anterior and posterior commissures (AC–PC line) (36). There are differences in histochemical and cytoarchitectonic properties between the pre-SMA and the SMA proper, but since there is no visible border between these two areas, a vertical imaginary plane passing through the anterior commissure and perpendicular to the AC–PC line is considered as the border (9, 30, 37). Instead of subdividing the SMA into the pre-SMA and SMA proper, the HCP subdivides the SMA into four parcellations: 6ma, SFL, 6mp, and SCEF; the first two parcellations are part of the terminations of the FAT. According to the HCP, from the SMA originates a medial bundle connected to the homologous contralateral SMA, a middle bundle descending to the basal ganglia and the corticospinal tract, and a lateral bundle, part of the FAT, connected to the IFG and insula (38). The HCP has also subdivided the dorsolateral prefrontal cortex into 13 areas; two of them, SFL and 8BL, are terminations of the FAT (28). The SFG is also connected to the inferior fronto-occipital fasciculus, the cingulum, and a callosal fiber bundle connecting the SFG bilaterally (5).

#### Inferior Frontal Gyrus

The IFG is delimitated superiorly by the inferior frontal sulcus, its posterior part inferiorly by the Sylvian fissure, and medially by the orbitofrontal gyri. The IFG is composed of three cortical regions: the pars orbitalis, the pars triangularis, and the pars opercularis, limited posteriorly by the precentral sulcus. Four major connections of the IFG have been identified and are represented by the FAT: the superior longitudinal fasciculus/arcuate fasciculus complex, the inferior fronto-occipital fasciculus, the uncinate fasciculus, and the callosal fibers connecting the IFG bilaterally (4).

#### Insula

The insula is hidden within the Sylvian fissure and is in continuity superiorly with the fronto-parietal opercular region and inferiorly with the temporal lobe. The central insular sulcus divides the anterior three short gyri from the posterior long gyri. The MI area lies in the posterior superior part of the short insular gyrus (39). The Human Connectome Project divided the insula in numerous parcellations (39) and found connections of the MI area with three SFG parcellations (6ma, 8BL, and SFL) through the FAT (29). The termination of the FAT in the insula has not been extensively studied, but Baker et al. (39) noted that a previously known network, the salience network (SN), has as nodes both FAT terminations and the anterior insula. The SN connects the fronto-insular cortex, composed of the ventrolateral prefrontal cortex and the anterior insula, to the anterior cingulate cortex (ACC) (40). This network, which also includes the amygdala, hypothalamus, ventral striatum, thalamus, and specific brainstem nuclei, is not only part of a functional network (41) but is also the only localization in the brain, jointly with BA 9 in the prefrontal human cortex, of the von Economo neurons (42). The fronto-insular cortex plays a role in interoceptive awareness of changes in homeostatic states, whereas the ACC generates relevant visceral, autonomic, behavioral, and cognitive responses. Through mutual interactions, these regions could respond to homeostatically relevant internal or external stimuli and enrich them with emotional weight (41). The salience network could mediate the switching between the processing streams of the default mode network and the central executive network during cognitively demanding tasks (40). This interconnection of the FAT with the anterior insula is also suggested by the similar spectrum of disorders that lesions to those regions cause. As the FAT, the anterior insula has been associated with progressive non-fluent aphasia PNFA, showing hypometabolism, atrophy (43), and gray matter damage (17) atrophy progression in large areas. This connection is also supported by the evidence that neurodegeneration in non-fluent variant (nfv) primary progressive aphasia (PPA) starts in a syndrome-specific epicenter and in the opercular region of the left IFG and then spreads to the most connected regions such as the SMA, insula, striatum, and inferior parietal regions (44).

#### Subcortical Connections

The SMA complex is connected to the limbic system *via* the cingulum and to the striatum (caudate nucleus and putamen) *via* short “U” association fibers and the superior longitudinal fasciculus I, cingulum, claustrocortical fibers, callosal fibers, corticospinal tract, frontal aslant tract, and frontostriatal tract (9). About 10% of the corticospinal fibers arise in the SMA proper, but no corticospinal fibers originate from the pre-SMA (45). The FAT is medial to the superior longitudinal fasciculus II (SLF II), which is orthogonal to the FAT, and lateral to the frontostriatal tract (FST) and claustrocortical fibers (CCF) (9, 13).

#### Regions of Interest for FAT Tracking

The FAT tracking is usually delineated by an axial “AND” region of interest (ROI) on the white matter of the SFG and a sagittal “AND” ROI on the white matter of the IFG (including the pars opercularis and triangularis) (22). The SMA ROI's anterior border is the anterior tip of the cingulate gyrus, while the posterior border is the precentral sulcus (34).

### Surgery-Related Deficits

Acute deficits reported immediately after surgery involving the FAT were aphasia, impairment of speech, self-initiated speech disorders, speech hesitancy, numerous pauses and delays during conversation, anomia, delays in naming and word finding difficulties, errors in verb generation tasks, perseverations, need for phonological cues, errors with reading, delay in counting, and simple calculations (35, 46–49).

Lesion of the FAT during tumor resection can result in peculiar deficits. In six patients with lesion close or inside the left FATs, only the last ones experienced transient impairment of speech. All patients recovered language function within 8 weeks (35). Young et al. (47) reported a case of a patient operated for a lower-grade diffuse glioma invading the dominant FAT, which was significantly disrupted in the post-operative diffusion tensor image (DTI). After transient symptoms, from post-operative day 4 to follow-up at 9 months after surgery, the patient still experienced fluent speech and intact naming/counting/sentence repetition. In one patient with brain tumor at the level of the left FAT, noun-based verb generation task and inverse task (i.e., verb-based noun generation) impairment, noted during intra-operatory stimulation of pre-SMA and left FAT, partially persisted 1 month after surgery, while performance on other language tasks remained acceptable. DTI confirmed left FAT damage and corona radiata partial damage, but left Broca's area was intact and the SMA/preSMA region was the only cortical region damaged (49). On five patients with left insular or frontal language-eloquent glioma, no one had a permanent surgery-related aphasia (46). A total of 19 patients with frontal glioma (14 left and five right) underwent awake surgery. Persistent speech initiation disturbances 3 months after the resection of a SMA glioma were noted only in one patient with left FAT disappearance. No post-operative speech disorders were observed after right-side surgeries (48).

Surgical access to frontal subcortical pathology has primarily been fulfilled *via* either transcortical or transcallosal routes. In order to reduce surgical injury to the white matter tracts and cortex, a tailored trans-sulcal para-fascicular corridor surgery to the frontal horn, third ventricle, and subcortical frontal lobe has been developed (13). Kocher's point (KP) represents the most used entry point to access the frontal horn of the lateral ventricle, and it relies exclusively on craniometric landmarks, not considering brain matter tracts, such as FAT, CCF, and SLF-II which are directly on KP trajectory. Kassam et al. purposely built and designed an optimized corridor to diminish subcortical surgical damage (50).

The role of the FAT in speech initiation was investigated through studies of electrical stimulation. Vassal et al. were the first to observe arrest of speech induced by stimulation of the left FAT during an awake resection of a left frontal lobe glioma in a right-handed patient without language deficits. The speech normalized again when the stimulation stopped (51). In another study by Fuji et al., FAT stimulation, on five right-handed patients, induced speech arrest in four patients and speech initiation delay in the other patient (52). Similar results were obtained by Kinoshita et al., who performed intra-operative electrical stimulation in 19 patients with frontal lobe tumors. Sixteen of these patients had speech arrest during the stimulation (48). The frontal aslant tract has been considered as part of the “negative motor network”; in fact, direct electrical stimulation over this tract causes movement arrest defined as negative motor response (53, 54).

During awake surgery of frontal tumors, direct cortical and subcortical electrostimulation (52, 55) combined with navigated tractography (51, 52) permitted to map and respect the FAT as a functional boundary.

Bizzi et al. observed that low-grade glioma (LGG) infiltration into the frontal intralobar tracts, including the FAT, may not always cause language deficits. In fact, LGGs tend to spare pars opercularis, the most eloquent area in the IFG, since infiltration of pars orbitalis and triangularis did not cause any language impairment (56). This could be explained by the adaptive plasticity of the frontal operculum and the presence of natural macroscopic (i.e., sulci) and microscopic barriers (i.e., cortical cyto-architecture) that may prevent the diffusion of the tumor into the pars opercularis (56).

The preservation of the FAT, despite acute post-surgical transient speech and motor disorders, permitted complete functional recovery within a few weeks after resection (51, 52, 55). Despite the preservation of the FAT, two patients out of 50, had permanent motor deficit, one due to injury to the supplementary motor area proper and one due to a partial injury of the corticospinal tract, but none of the patients experienced permanent speech disturbance after tumor removal (55).

### Roles in Verbal Fluency

Verbal fluency is a cognitive function that helps information retrieval from memory. Semantic fluency is tested by asking to generate words belonging to given categories (e.g., names of animals), while phonemic fluency is tested by asking for words beginning with a given letter, usually F, A, and S (57).

Microstructural abnormalities of the FAT were significantly associated with verbal fluency deficits measured by mean length of utterance and words-per-minute tasks in patients with primary progressive aphasia. Catani et al. found no correlations between the FAT and measures of overall language impairment, grammar deficit, repetition or single word comprehension (measured, respectively by Western Aphasia Battery Aphasia Quotient, Northwestern Anagram Test Western Aphasia Battery—Repetition and Peabody Picture Vocabulary Test) (15). Alteration of the left FAT is correlated only with nfv in PPA (15, 16). This suggests a dissociation between verbal fluency and semantic processing functions, which relay, respectively on the FAT and on the uncinate fasciculus (15, 17). Mandelli et al. results strongly suggest that neurodegeneration in nfv-PPA starts in a syndrome-specific epicenter in the dorsal portion of the opercular region of the left IFG and then spreads most significantly to the SMA through the FAT (44).

In chronic post-stroke aphasia speech, fluency was uniquely correlated with left motor cortex and underlying white matter (including the anterior section of the arcuate fasciculus and the frontal aslant tract) (18, 58, 59). Damage to FAT in chronic aphasia due to left-hemisphere ischemic stroke correlated with both semantic and phonological fluencies (60).

In a patient with crossed aphasia, cholinergic potentiation and audiovisual repetition–imitation therapy improved language deficit through modifications in the right FAT and the right direct segment of the arcuate fasciculus (61).

In multiple sclerosis patients, verbal fluency is significantly correlated with mean fractional anisotropy (FA) in bilateral frontal aslant tract (62, 63).

In adults with a history of very preterm birth worse verbal fluency than controls is correlated to FAT properties and laterality (64). No association between the frontal aslant tract and verbal fluency was found in 29 right-handed, healthy university students; however, lexical decision was correlated with FAT laterality (32).

Single-photon emission computed tomography and functional near-infrared spectroscopy suggested that FAT may play a crucial role in word retrieval difficulty in acute thalamic stroke survivors; furthermore, SMA may contribute to improve word retrieval difficulty (65). No correlation between FAT and apraxia of speech (66) or syntax (67) has been noticed. Naming recovery in patients with aphasia after a left hemispheric stroke also showed no correlation with FAT (68). In subthalamic nucleus deep brain stimulation, the most reported adverse effect is verbal fluency impairment, but it could be not associated with the damage of fiber pathways along the electrode trajectories, including the FAT (69).

Speech fluency can be measured by different tests, such as the Western Aphasia Battery-Revised (WAB-R) fluency subtest and words per minutes (WPM) test. The WPM and WAB fluency are related, but not redundant, measures of fluency. The WPM and WAB fluency scores highlight the role of the FAT in verbal fluency (15, 58). Patients with FAT disconnection showed significantly worse phonemic fluency test scores (70). Low scores in Brief Language Assessment for Surgical Tumours patients' articulatory agility task, which requires reciting utterances as rapidly as possible (e.g., 50, 50, 50…), are associated with pathologies overlapping with the territory of the FAT (71). In nfvPPA, the FAT microstructural properties were associated with the number of distortion errors per hundred words that patients made in spontaneous speech during the WAB spoken picture description task (17).

### Roles in Lexical Decision

Sierpowska et al. firstly suggested a relationship between a FAT damage and lexical retrieval deficits. The authors performed awake surgery to resect a left frontal tumor and observed, at the time of tumor resection and at the left FAT intraoperative electrical stimulation, that the patient, while performing a noun-based verb generation task, applied a morphological derivation rule to the given nouns to form new inexistent verbs instead of retrieving proper existing verbs (49). Pre-operative and post-operative fMRI analyses revealed that Broca's area and preSMA were both activated during the verb generation task. Indeed several studies have established the role of Broca's area in language production, lexical retrieval, and/or selection of semantic knowledge and grammatical/morphological processing (49, 72–77), the role of the SMA in speech initiation, coordination and monitoring, and articulatory abilities (78–81), and the role of the pre-SMA in linguistic production (15). All of these regions are cortical terminals of the FAT which, in the post-operative tractography DTI, was confirmed to be damaged. After surgery, the patient had good abilities in semantic decisions, past and present tense forms, and phonological production; verbal fluency and working memory were instead considerably affected along with the performance in the noun-based verb generation task and also in the inverse task of verb-based noun generation. In another case study, Chernoff et al. considered two patients, one underwent surgical resection of a left frontal glioma and the other one underwent left anterior temporal and hippocampal resection (82). In the first patient, the post-operative DTI evaluation revealed microstructural impairment of the left FAT and clinically dysfluent speech in complex sentences without impairment in lexical access. On the contrary, the second patient presented impairment of the left inferior longitudinal fasciculus and word finding difficulties without dysfluent speech. Other language functions were not affected in any patient. To further investigate the role of the FAT in the mediation process from sentence planning to lexical access, the authors performed a second case study of a patient undertaking awake surgery to remove a left frontal brain tumor. During the surgery, the patient was given a task consisting of generating a sentence to describe the spatial relation of a target marked shape (the grammatical subject of the sentence) with the shape above or below it. In the course of the intra-operative task execution, stimulation of the left FAT generated a prolonged inter-word time at the beginning of syntactic phrases, but inter-word duration within phrases was either not affected by stimulation or reduced, along with the sentence's total extent and intra-word duration. Given this result, the authors suggested a potential role of the left FAT in integrating grammatical information with the sentence structure, thus introducing the “Syntagmatic Constraints On Positional Elements” hypothesis (83). These evidences lead Corrivetti et al. to retrospectively analyze functional language maps of both white and gray matter regions obtained in 17 patients undergoing awake surgery for left frontal lobe glioma resection. The conclusion of this study was that motor–speech responses and lexico-semantic responses are both functions conveyed by the FAT; specifically, the lexico-semantic role belongs to the anterior FAT, while the motor–speech function is attributable to the posterior FAT (84). In contrast with these findings, a recent study considering 20 patients with a left-hemisphere stroke located in the frontal lobe did not show any association between a lower FAT volume and lower conceptual or lexical selection abilities. The behavioral assessment was measured using the sentence completion task to evaluate conceptual and lexical selection and the picture–word interference task to specifically evaluate the lexical selection. The authors tried to explain this variance from previous findings, confirming the idea of the FAT involvement in these functions but assuming a possible reorganization of the FAT during the post-stroke recovery period (85). Finally, Vallesi et al. investigated, in a group of 29 healthy university students, the correlation between macrostructural and microstructural properties of the FAT, evaluated though the utilization of DTI indices and the lexical decision processes. The latter were evaluated through a lexical decision task, in which the students had to estimate if the letter strings provided were real Italian terms or invented ones, and the color and shape discrimination task, in which they had to specify the color and the shape of the presented stimulus. The result of this study was the evidence, for the first time, of a positive association between left lateralization of the FAT and faster lexical decision latency. However, no correlation was observed between the lateralization indices of the FAT and verbal fluency (32).

### Roles in Stuttering

Stuttering is a childhood-onset speech fluency disorder that sometimes persists into adulthood, consisting in sound prolongations and repetitions along with interrupted words regardless of articulatory features (86). Recently, persistent developmental stuttering has been associated with anatomical abnormalities and lower activation of the IFG and the ventral premotor cortex (PMv) (87, 88). This theory is aligned with the results obtained by Chesters et al. who, after applying direct current stimulation on the left IFG/PMv, observed an improvement of speech fluency in people with stuttering (89). Starting from these evidences, recent studies have investigated the role of the FAT in speech fluency in people with stuttering. Among these, Kronfeld-Duenias et al. grouped 15 adults with persistent developmental stuttering and nine healthy controls and then analyzed through tractography the volume and diffusion properties of the FAT. As a result, increased mean diffusivity in the left FAT was observed in the group with stuttering compared with controls. Moreover, a negative association was found between diffusivity values and speech rate and fluency in the individuals with stuttering. To evaluate the occurrence of stuttering, the authors used an interview about a neutral topic and a reading task, and the severity was instead assessed with the Stuttering Severity Instrument-III (90, 91). In another study considering eight patients with no pre-operative stuttering, Kemerdere et al. showed that direct electrical stimulation of the left FAT, conducted throughout awake surgical resection of a left frontal glioma, induced intra-operative transient stuttering. No patient experienced post-operative stuttering though during tumor resection, and the FAT was preserved in all cases. In two patients, minor speech initiation disorders persisted after surgery (92). Based on these results and on the studies observed above, the authors identify the disconnection of the cortico-subcortical circuit, including the FAT that supports the speech motor control, as a potential etiopathogenesis of stuttering. Recently, Neef et al. recruited a group of 31 adults with stuttering and a second group of 34 healthy controls (93) and found impaired white matter integrity of the right FAT in the group with stuttering. Moreover, a stronger connectivity of the right FAT was positively associated with stuttering severity, suggesting an enhanced speech–motor suppression mechanism in stuttering.

### Roles in Executive Functions

Taking the premise that the FAT is a white matter bundle connecting secondary motor areas, in particular, the Broca's area with the SMA and the pre-SMA regions were shown to be involved, respectively in the online control and in the planning of simple reaching and grasping actions (94).

The SMA syndrome is a well-known neurosurgical disturbance that may appear after surgery has been performed in the unilateral SMA region. This syndrome is defined by a transient inability to initiate contralateral voluntary movements which typically spontaneously disappear within 3 months, except for the incapability to alternate bimanual gestures that is often irreversibly affected (95). In six patients operated through surgical excision of low-grade glioma located in the SMA region, no statistically significant association was found between recovery time and damage of white matter tracts contiguous to the SMA, including the FAT, FST, and pyramidal tract, except for the cingulum (96). The mechanism of functioning restoration after surgical damage of the FAT is undiscovered, but it likely involves plasticity of the cortical language network and recruitment of the contralateral hemisphere, possibly through transcallosal fibers (47). In fact, right FAT has also a role in recovery after left FAT lesion-associated speech deficit as suggested by the evidence that cholinergic enhancing, alone or integrated with a model-based aphasia therapy, promotes improvements in aphasia by inducing structural plastic changes in right FAT. Baker et al. hypothesize that a possible mechanism involved in the recovery from SMA syndrome may be represented by not equivalent bonds between contralateral motor areas by supporting interhemispheric connectivity (10). For this reason, commissural fibers from the contralateral SMA region should be preserved in order to facilitate the resolution of transcortical motor aphasia that typically occurs after resection of SMA lesions (52).

Budisavljevic et al. suggested, for the first time, a potential role of the FAT in the visuo-motor process that supports movement planning and feedback control during hand movement vs. a target object. To support this idea, the authors used DTI to analyze the microstructural organization of the bilateral FATs in 32 right-handed, healthy participants who were asked to perform a reach to grasp task and a reach task vs. a target object. As a result, a higher anisotropy of the bilateral FAT resulted to be associated with a more efficient visuo-motor processing and more stable paths, in particular, with lower acceleration and deceleration amplitude ranges of reach and reach-to-grasp movements (22).

Afterwards, the hypothesis of a potential FAT involvement in the neurological mechanisms underlying visuo-motor integration was supported by two other studies. In the first one, Serra et al. enrolled 23 patients with Alzheimer's disease (AD) and conducted a probabilistic tractography analysis and examination of the bilateral FATs FA. Not only the mean FA resulted to be significantly lower bilaterally in patients with AD compared to healthy subjects (HS) but also the FA in the right FAT resulted to be positively associated with patients' performance at copy of drawings and copy of drawings with landmarks tests (that evaluate constructional praxis) and Raven's colored matrices (that evaluates visuo-spatial logical reasoning) (23). In the other study, Tsai et al. considered 10 adults with amblyopia and showed a lower mean FA in in the left FAT compared to HS (97). Considering that both apraxia and amblyopia are associated to visuo-motor integration deficits—in fact, constructional apraxia is defined as the inability to reproduce spatial patterns due to an impairment of visuo-spatial analysis and integration with motor planning and skills (25), while amblyopia was reported to be associated with visuo-motor defective abilities in tasks demanding precision and speed (98, 99)—these results support the idea that the FAT may have a role in these processes. Moreover, Budisavljevic et al., relying on the observed association between movement deceleration and the bilateral FATs and supported by previous subcortical stimulation studies of the white matter corresponding to the nowadays FAT producing a deceleration (100) or complete interruption (101) of both hands movements, also suggested for the first time a potential involvement of the FAT in the inhibitory control of motor pathway (22). This idea is supported by studies of fMRI, DWI, TMS, direct cortical/subcortical stimulation, and electrocorticography showing that the right IFG and the right SMA and pre-SMA, both interconnected by the FAT, play a role in the neural motor network in conducting inhibitory regulation processes (24). In particular, these regions have been described by Aron et al. as parts of a cortico–basal ganglia–thalamic–cerebellar circuit (102) where, more specifically, both the right IFG and the pre-SMA connect to the subthalamic nucleus and play a role, respectively, in suppressing cortical output and resolving conflicting behaviors (103, 104). Motor inhibition has been evaluated through go/no-go and stop signal experimental models, where a powerful response is launched at first (go trial), and then it must be supplanted when a stop signal appears (stop trial) (105–108).

Based on these evidences, Dick et al. assumed that the FAT is a component of the cortico–basal ganglia–thalamic–cerebellar anatomical–functional circuit described above and plays a role in executive functions, especially in the programming and coordination of sequential motor movements through a selection among motor plans that compete for the same motor resources. The authors, in accordance with computational models of inhibitory regulation for speech and for manual actions, assume that this function is present bilaterally but is differently specialized between the two hemispheres: the left FAT is specialized for speech programming and controls the articulatory apparatus, while the right FAT is specialized for general action and for visuo-motor integration, regulating the manual/limb and the oculomotor systems (24).

### Roles in Oro-Facial Movements

Foix–Chavany–Marie syndrome (FCMS) is a rare syndrome, usually caused by bilateral lesions of the anterior operculum, for this reason it is also known as opercular syndrome. FCMS is a form of pseudobulbar palsy, clinical manifestations range from severe articulatory disorders to mutism (109), limb weakness and bowel and bladder incontinence, but with preservation of involuntary reflex motor movements of the affected muscles, such as smiling or crying (110). Symptoms usually recover over a matter of days or over a timespan of months (111). Surgical damage of connections between FAT and arcuate fasciculus, and the right pars opercularis caused post-operative FCMS. For this reason a trans-opercular approach to insulo-opercular gliomas can generate FCMS (112). In a patient with opercular syndrome, a volume reduction was noticed in the primary motor cortex, SMA, posterior portion (BA6) of the operculum and white matter of the frontal lobe, with a left prevalence, including the CC, AF, SLF, FAT, and CST (109, 113).

Signs of spastic and atrophic bulbar palsy are also present in amyotrophic lateral sclerosis. Disease duration in amyotrophic lateral sclerosis patients is associated with atrophy in the cortical terminations of left FAT and the right precentral gyrus (114).

### Roles in Working Memory

Working memory comprises a complex brain system that maintains information for periods of time going from seconds to minutes and allows processing of information for future goal-directed behavior and complex cognitive tasks (115). Recent studies showed that the FAT can also have implications in working memory performances. Rizio et al. performed neuropsychological tasks and DTI in two groups of adults, one group under 35 years old and the other over 59 years old, with the aim to evaluate age-related changes in speed, language, working memory, episodic memory, and inhibitory control. Working memory evaluation included spatial working memory and both backward and forward digit span. In both groups, age was a predictor of working memory, but only in the older group the integrity of bilateral FAT and left SLF/AF, evaluated through FA, was a marginal predictor of working memory ability (19). This result paved the way to other studies that investigated the FAT functional implication in working memory. Varriano et al. proposed an extended definition of the FAT (“exFAT”) that ends further anteriorly into the SFG. The authors evaluated its volume and laterality in four groups of participants selected from a total of 900 subjects according to their performance in language and working memory tasks. The authors observed that the exFAT was not lateralized in any group; there were statistically significant differences instead in the volume of the left exFAT between the groups of best performers and worst performers in the language task and of the right exFAT between top performers and bottom performers for 2-back working memory task, but not for the 0-back working memory task (21). In these *n-*back tasks, a series of visual stimuli appears, and the subjects were asked for each stimulus as to whether it corresponds a stimulus *n* trials ahead (20). The FA of the right FAT was also found to be associated with better visual memory performance in the delayed matching to sample task in a study considering 39 healthy brothers of autism spectrum disorder (ASD)-affected boys (116). This task consists on presenting a stimulus to the subject to make them memorize it, and after a delay, the stimulus is presented again but with other stimuli, and the subject has to choose the right one (117). These evidences suggest that the FAT is another tract to be considered during tasks performed in awake surgical resection of tumors located in the right frontal lobe in order to preserve the working memory function. The working memory was particularly examined in individuals with right non-dominant frontal tumors so far, using intraoperative tests, such as the digit span test for verbal working memory and the 2-back test for spatial working memory (55, 118).

Finally, in a study conducted by Chen et al., mean diffusivity of the bilateral FAT, along with the bilateral superior longitudinal fasciculus, was observed to be significantly associated to fluid intelligence (119). Fluid intelligence is defined as an innate capability, independent from experience and education, that allows one to make logical reasons and decisions to solve problems and respond to complex and unpredictable situations. Since this ability is also linked with working memory, executive functions, and attention, this result is aligned with the studies mentioned above (120).

### Roles in Social Community Tasks

In 2014, Catani and Bambini have proposed a five-levels model for social communication based on results of functional and anatomical neuroimaging studies in humans. For each level, including informative actions, communicative intentions, lexical and semantic processing, syntactic analysis, and pragmatic integration, they identified the correlated white matter tracts. On the bases of the regions and relative functions connected by the FAT, the authors associated this tract to the communicative intentions level (level 2), suggesting a role in identification and expression of communicative purposes (121). Recently, a relationship has been described between social communication deficits in ASD and FAT integrity, evaluated through FA. ASD is a neurological and developmental disorder with social communication deficits and social reciprocal interaction impairment as core symptoms, and the diagnosis is clinical according to the Diagnostic and Statistical Manual of Mental Disorders (DSM–5) diagnostic criteria (122, 123). These problems may be seen with various grades of severity using different scales, of which the most important are the Autism Diagnostic Interview—Revised and the Social Responsiveness Scale for subjects above 18 months, the Social Communication Questionnaire, and the Child Behavior Checklist (CBCL) for individuals above 4 years old (124). Lo et al. firstly identified that the microstructural integrity of the bilateral FAT was decreased in a group of 62 right-handed boys with ASD compared to a group of 55 normally developing boys. Moreover, the FA values resulted to be significantly associated with the severity of socially related communication and interaction deficits in the ASD group (125). The same results were obtained later in another study that had also shown a reduction of the bilateral FAT integrity in unaffected siblings of subjects with ASD (116). Based on these results, Lo et al. tried to identify intermediate phenotypes of social communication deficits in ASD, taking into consideration three different groups: 30 boys with ASD, 27 healthy brothers of individuals with ASD, and 30 normally developing boys. According to previous results, the FAT integrity was reduced both in ASD subjects and in the unaffected siblings. Moreover, the reduction was also associated with the social communication scores (126). These findings suggest that the FAT may potentially contribute to the neural processes involved in social communication deficits in ASD.

### Roles in Attention

Attention-Deficit Hyperactivity Disorder (ADHD) is a complex and heterogeneous brain developmental condition associated with excesses levels of hyperactivity and inability to concentrate (127). The clinical diagnosis is made according to the DSM-5 criteria (123), but ADHD tendency can be assessed through the Conner's Comprehensive Behavior Rating Scale in children and the Conner's Adult ADHD Rating Scales in adults (128). Garic et al. observed, for the first time, a relationship between left laterality of the FAT and attention problems in children (34). In a group of 70 subjects younger than 19 years old, the left laterality of the FAT predicted greater attention problems (measured *via* CBCL) and lower executive function abilities (measured *via* the Behavior Rating Inventory of Executive Function). This result is aligned with the previous structural and functional neuroimaging studies showing that right IFG and pre-SMA alterations are also associated with impaired executive function and ADHD (129–131).

### Roles in Music Processing

Since the IFG and the motor cortical areas have been shown to contribute to music–syntactic and rhythm processing, the FAT, connecting these areas, may also be involved in music processing (132). The first evidence comes from a study conducted in a group of 42 right-handed stroke patients. Structural impairment of different white matter tracts, including the FAT, resulted to be associated with post-stroke non-recovered amusia. To evaluate the music perception of the patients, the authors used the Montreal Battery of Evaluation of Amusia (133). According to the other FAT functions mentioned above, the role of the FAT in music processing and perception may be, in particular, related to its role in attention and working memory, both of which are useful to allow online correlation and differentiation of subsequent sounds.

## Conclusion

**Figure 4:**
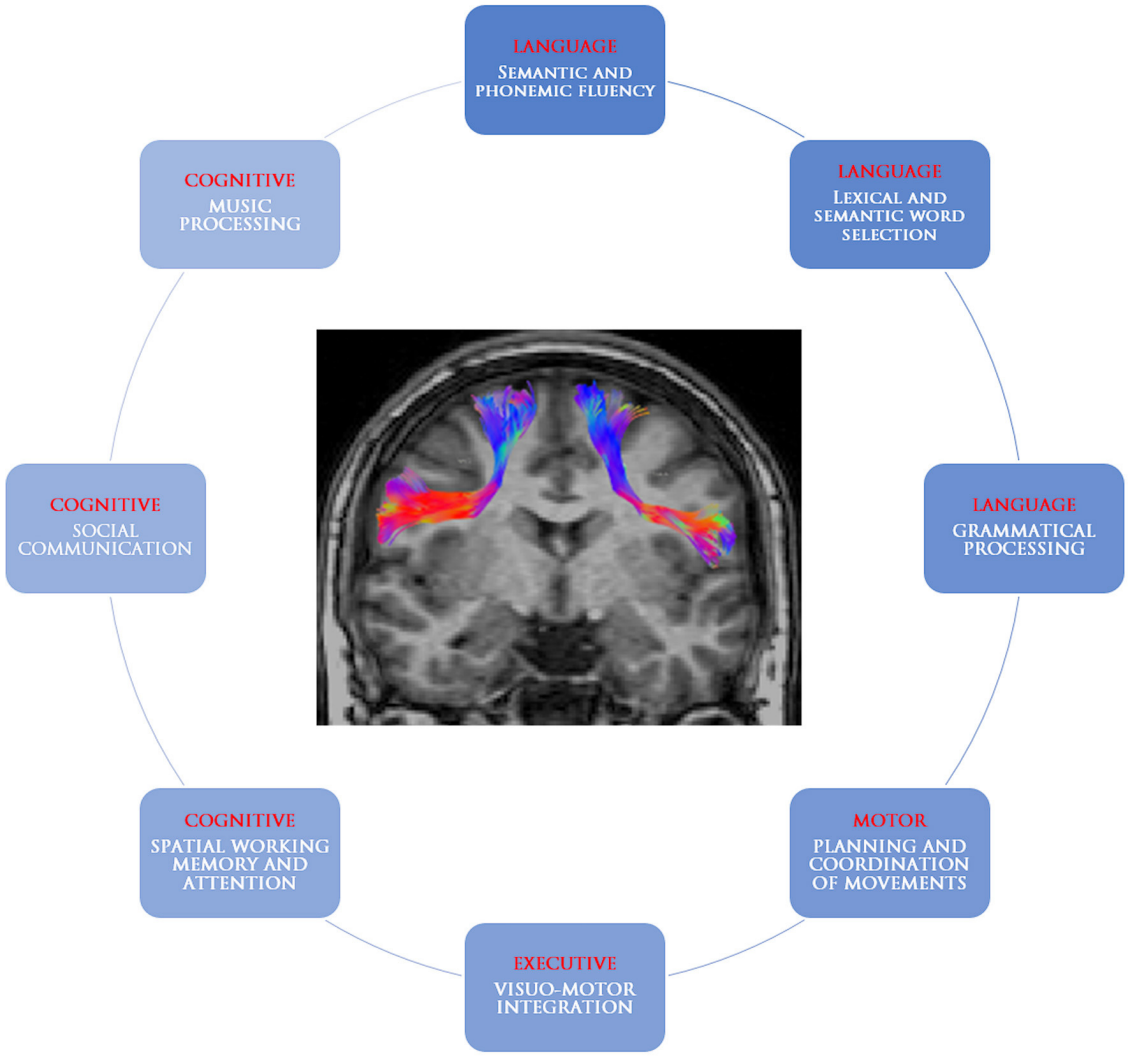
Graphical representation of the frontal aslant tract and its putative roles.

The frontal aslant tract is a recently identified white matter tract connecting the supplementary motor area complex and the lateral superior frontal gyrus to the ipsilateral inferior frontal gyrus and the anterior insula. The present review retrieved studies suggesting its involvement in speech and language functions (verbal fluency, initiation and inhibition of speech, sentence production, and lexical decision) as well as executive functions, visual–motor activities, orofacial movements, inhibitory control, working memory, social community tasks, attention, and music processing (Table 1, Figure 4). The acquired knowledge on the FAT anatomical connectivity and its functional roles may raise awareness in the neurosurgical community to set up their practical applications in routine surgical activities and to pose future foundation for intraoperative stimulation research studies.

**Table 1:** Summary of putative frontal aslant tract functions and useful assessment tests.

**Roles**	**Specific functions**	**Evaluation tests**
Language	Verbal fluency	Phonemic fluency tests (FAS) Mean length of utterance Western Aphasia Battery-Revised (WAB-R) fluency subtest Words per minutes (WPM) test
	Control of the articulatory apparatus	Interview about a neutral topic Reading task Stuttering Severity Instrument (SSI)
	Lexical and semantic word selection	Noun-based verb generation task Verb-based noun generation task Sentence completion task Picture-word interference task Lexical decision task
	Grammatical processing	Sentence generation task
Motor and executive functions	Visuo-motor integration	Reach to grasp task Reach task vs. a target object
	Constructional praxis	Copy of drawings test Copy of drawings with landmarks test Raven's Colored Matrices
	Inhibitory regulation of speech and motor actions	Go/No Go trial Stop-Signal trial
	Executive function abilities	Behavior Rating Inventory of Executive Function (BRIEF)
Working memory	Verbal, spatial and visual working memory	Backward and forward digit span test n-back working memory task Delayed Matching to Sample (DMS) task
Social community and attention tasks	Identification and expression of communicative purposes and ability to concentrate	Autism Diagnostic Interview-Revised (ADI-R) Social Responsiveness Scale (SRS) Social Communication Questionnaire (SCQ) Child Behavior Checklist (CBCL)
Music processing	Online correlation and differentiation of subsequent sounds	Montreal Battery of Evaluation of Amusia (MBEA)

## Data Availability Statement

The original contributions presented in the study are included in the article/Supplementary Material, further inquiries can be directed to the corresponding author/s.

## Author Contributions

ELC, DE, and EG: conception and design, and drafting the article. ELC: approved the final version of the manuscript on behalf of all authors. PF, MB, and GS: administrative/technical/material support and study supervision. All authors: acquisition of data, critically revising the article, and reviewed submitted version of manuscript.

## Conflict of Interest

The authors declare that the research was conducted in the absence of any commercial or financial relationships that could be construed as a potential conflict of interest.

## Supplementary Material

Supplementary Table 1 Studies included in the qualitative synthesis of the systematic literature review according to PRISMA guidelines.
